# Partial Discharge Fault Diagnosis in Power Transformers Based on SGMD Approximate Entropy and Optimized BILSTM

**DOI:** 10.3390/e26070551

**Published:** 2024-06-27

**Authors:** Haikun Shang, Zixuan Zhao, Jiawen Li, Zhiming Wang

**Affiliations:** Key Laboratory of Modern Power System Simulation and Control & Renewable Energy Technology, Ministry of Education, Northeast Electric Power University, Jilin 132012, China; 2202200139@neepu.edu.cn (Z.Z.); 2202300208@neepu.edu.cn (J.L.); 2202300311@neepu.edu.cn (Z.W.)

**Keywords:** partial discharge, power transformer, symplectic geometry mode decomposition, approximate entropy, bidirectional long short-term memory

## Abstract

Partial discharge (PD) fault diagnosis is of great importance for ensuring the safe and stable operation of power transformers. To address the issues of low accuracy in traditional PD fault diagnostic methods, this paper proposes a novel method for the power transformer PD fault diagnosis. It incorporates the approximate entropy (ApEn) of symplectic geometry mode decomposition (SGMD) into the optimized bidirectional long short-term memory (BILSTM) neural network. This method extracts dominant PD features employing SGMD and ApEn. Meanwhile, it improves the diagnostic accuracy with the optimized BILSTM by introducing the golden jackal optimization (GJO). Simulation studies evaluate the performance of FFT, EMD, VMD, and SGMD. The results show that SGMD–ApEn outperforms other methods in extracting dominant PD features. Experimental results verify the effectiveness and superiority of the proposed method by comparing different traditional methods. The proposed method improves PD fault recognition accuracy and provides a diagnostic rate of 98.6%, with lower noise sensitivity.

## 1. Introduction

Electric power transformers play a pivotal role in the power system [1]. They are responsible for transmitting high voltage over long distances and stepping it down to a suitable low voltage for distribution. This conversion not only reduces energy losses during long-distance transmission but also ensures the fulfillment of various electricity demands for households, industries, and commercial facilities [2]. As a result, the efficient operation of power transformers is crucial for maintaining the stability and reliability of the power system.

Partial discharge (PD) is a phenomenon that occurs in the insulation system of the power transformers, which may pose serious risks to the insulation performance [3]. This phenomenon can lead to the damage of insulation materials, resulting in a decline in insulation performance and an increased risk of equipment failure. The arcs and thermal effects generated by PD may cause insulation breakdown. The prolonged existence of PD can gradually damage the insulation system of the transformer, reducing its operational stability and reliability [4]. Therefore, the timely monitoring of PD is of vital significance to ensure the normal operation and prolong the lifespan of power transformers.

Analyzing the correlation between PD patterns and specific faults can facilitate the early detection of potential issues [5]. This aids in implementing targeted maintenance measures, thus enhancing the maintenance efficiency. Feature extraction enables the analysis of characteristics within the PD signals, constituting a crucial step in the diagnosis of insulation faults in transformers. It directly influences diagnostic effectiveness [6].

In the field of PD fault diagnosis, various feature extraction methods have emerged, including time-frequency domain analysis [7], wavelet transform [8], empirical mode decomposition (EMD) [9], variational mode decomposition (VMD) [10], and so on. Time-domain feature extraction focuses on waveform morphology, amplitude, etc., suitable for capturing transient characteristics. However, for complex non-stationary PD signals, there may be significant information loss. Frequency-domain feature extraction is based on the spectral properties of PD signals, such as spectral peak and bandwidth, revealing the frequency distribution of discharge signals [11]. Yet, it disregards the temporal information of the signals. wavelet transform decomposes signals into different frequency components, analyzing signals in both time and frequency domains, suitable for multi-scale feature extraction. It excels at describing changes in different frequency components but selecting appropriate wavelet basis functions can be challenging [12]. EMD is an adaptive decomposition method that separates signals into intrinsic mode functions (IMFs), each with specific frequency characteristics [13]. EMD is suitable for nonlinear and non-stationary signals, effectively capturing the transient features of signals; however, EMD may suffer from mode mixing when dealing with high-frequency noise. It involves substantial computation and has stability issues. VMD decomposes signals into modulation components, overcoming the mode-mixing limitations in EMD [14]. VMD is also applicable to nonlinear and non-stationary signals. It can effectively distinguish different frequency components. Nevertheless, the parameter selection for VMD can be relatively complex, involving significant computation.

Symplectic geometry mode decomposition (SGMD) is based on the theory of symplectic geometry and represents multi-mode data as points on a symplectic manifold [15]. The symplectic manifold represents a unique geometric structure that preserves the nonlinear properties and manifold structure of data. SGMD conducts decomposition on the symplectic manifold, breaking down multi-mode data into a set of modes and capturing distinct features of the data. It retains the nonlinear structures and mode relationships through symplectic manifold representation, thus overcoming the traditional modal aliasing issues in EEMD and pre-set parameters in VMD. Pan et al. [16] introduced the SGMD algorithm and applied it to rotating machinery compound fault diagnosis. Compared with EEMD, LCD, and wavelet methods, the results of the simulation and experimental signals indicate that SGMD provides enhanced diagnostic effectiveness for compound faults in rotating machinery. A novel signal decomposition method based on SGMD has been proposed for extracting features of lubricating oil debris [17]. SGMD offers the capability to adaptively reconstruct signals. Simulation results demonstrate its effective extraction of debris features, surpassing the decomposition abilities of EMD or wavelet. In summary, a large amount of existing research indicates that SGMD has been widely used in the industrial field. Due to its comprehensive foundation in symplectic geometry theory, it exhibits outstanding advantages in problem-solving compared to traditional decomposition methods. Moreover, the application of SGMD in the field of transformer PD diagnosis has not been reported. Therefore, this paper attempts to utilize SGMD for the PD signal feature extraction.

PD signals have non-stationary and nonlinear characteristics. With SGMD decomposition, it is difficult to extract the features representing the complexity and irregularity of the signal. In order to further extract comprehensive features, information entropy is chosen to measure the uncertainty of PD signals. Information entropy is a significant concept in information theory to measure the uncertainty or disorder of a random variable [18]. It finds widespread applications across various domains. Among these applications, approximate entropy (ApEn) is a metric to gauge the complexity and irregularity of time series [19]. It yields particularly effective results in analyzing nonlinear dynamic systems, revealing their nonlinear characteristics and aiding the exploration of chaotic properties. Notably, ApEn exhibits robustness against noise, enabling it to mitigate the impact of noise. Presently, ApEn has demonstrated notable efficacy in fields such as biomedical research [20], mechanical engineering [21], aviation [22], and more. This study attempts to utilize ApEn for quantifying the extracted features from the PD signals in transformers.

As the second step of transformer PD fault diagnosis, pattern recognition directly influences diagnostic outcomes [23]. The SGMD ApEn feature extraction method proposed in this article needs to be combined with a suitable classifier to achieve the goal of improving diagnostic accuracy. The theory of deep learning holds immense potential in the field of pattern recognition [24,25]. By constructing multi-layer neural network structures, deep learning can autonomously learn abstract features from data, facilitating efficient feature representation and pattern classification [26]. In domains such as images [27], speech [28], and natural language processing [29], deep learning has achieved remarkable success. This signifies the substantial scope for deep learning to revolutionize pattern recognition, thereby contributing to enhanced accuracy in classification, detection, and prediction, offering innovative solutions across various domains. The different PD signals of transformers have similar characteristics, which may have an important impact on the faults’ identification. As a novel type of machine learning method, deep learning can obtain the separability representation of various types of samples adaptively; therefore, this article attempts to use deep learning theory for PD recognition. Recurrent Neural Networks (RNN) [30] in deep learning have been widely applied to fault recognition in various domains such as meteorology [31], computer science [32], and medicine [33]. Long short-term memory (LSTM) [34], a variant of RNN, addresses the vanishing gradient issue. Bidirectional long short-term memory (BILSTM) [35], an improvement of LSTM, introduces a bidirectional time structure to capture information at each node in a time series. BILSTM achieves higher prediction accuracy by extracting information comprehensively. In this paper, on the foundation of BILSTM, Adaboost ensemble learning technology is introduced to enhance recognition capability. The golden jackal optimization (GJO) is employed for the parameter configuration in BILSTM. The optimized model is applied for transformer PD fault diagnosis. A performance comparison of different diagnostic methods is shown in Table 1.

This work proposes a novel approach for transformer PD fault diagnosis, combining feature extraction and pattern recognition. Firstly, PD signals are collected in an experimental setup. Afterward, the signals are decomposed using SGMD to obtain SGCs. Effective SGCs are selected using similarity theory, and their ApEn values are calculated as PD features. Finally, the PD features are sent into an optimized BILSTM for fault diagnosis. The effectiveness and practicality of the proposed method are validated through the simulation and experimental data.

The organizational structure of this paper is as follows. The principles of the relevant algorithms are detailed in Section 2. Section 3 validates the effectiveness and superiority of the algorithms using simulated signals. Section 4 presents a transformer PD fault diagnosis model based on SGMD and BILSTM. Section 5 concludes the paper.

## 2. Algorithm and Principles

The BILSTM model consists of an input layer, a forward LSTM, a backward LSTM, and an output layer [36]. This model gives each output node complete bidirectional temporal information.

However, the individual BILSTM network is found to be difficult to simultaneously model the multiple faults. In order to improve the model’s ability to represent complex multi-class data features, the ensemble learning technology is introduced.

In this paper, the Adaboost algorithm trains the BILSTM network in an iterative manner. After iterations, the BILSTM models that focus on different data features are obtained. In each iteration, the training data are generated by probabilistic random sampling. After training, the weight *β* is generated according to the error rate of each model [37]. The sample probability weight of the training data is adjusted to change the next round of distribution. Finally, all BILSTM models are combined according to the weights [38].

**Figure 1:**
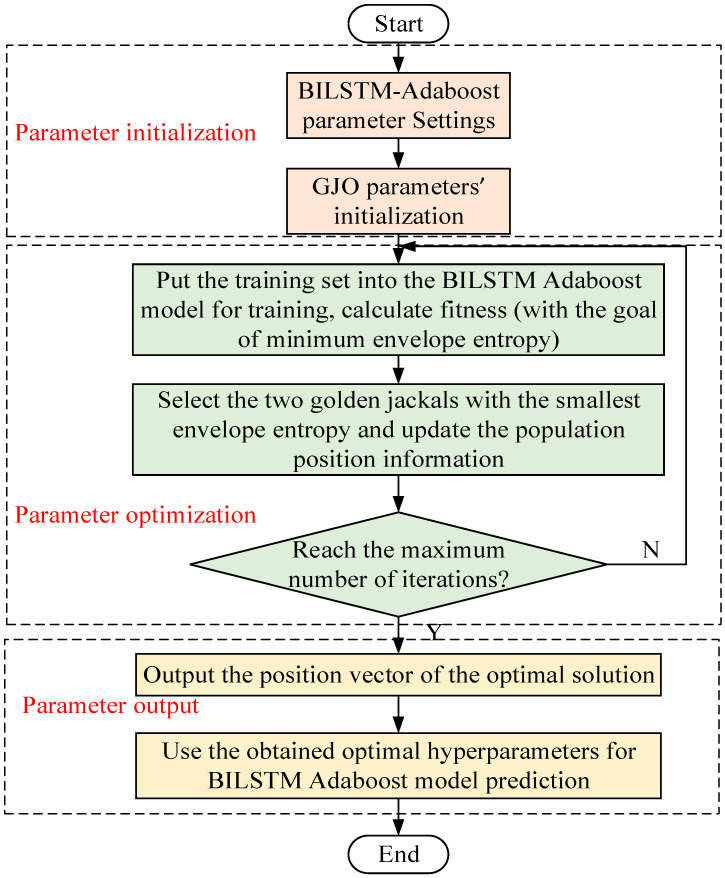
The flowchart of GJO-BILSTM-Adaboost.

In recent research, the parameters in neural networks are commonly selected by various optimization methods, which may suffer from the problems of slow convergence speed and numerous iterations [39]. In this work, the GJO algorithm is introduced for parameter optimization, with a good global search ability and high convergence speed. The minimum envelope entropy is selected as the fitness function for the GJO algorithm. The flowchart of GJO-BILSTM-Adaboost is shown in Figure 1.

## 3. Simulation Analysis

This article utilizes simulated signals to validate the effectiveness and practicality of the SGMD decomposition algorithm. PD signals from power transformers can be represented using an exponential damped oscillation model, as shown in Equations (1) and (2) [40].

(1) $$S_{1}(t)=K(e^{-\alpha_{1}t/\tau }-e^{-\alpha_{2}t/\tau })\cdot \sin(f_{c}t)$$

(2) $$S_{2}(t)=Ke^{-\alpha_{1}t/\tau }\cdot \sin(f_{c}t)$$

where *K* represents the signal amplitude, measured in V; *α*_1_ and *α*_2_ are the attenuation parameters; *τ* is the attenuation period, measured in ms; *f_c_* is the oscillation decay frequency, measured in MHz.

**Figure 2:**
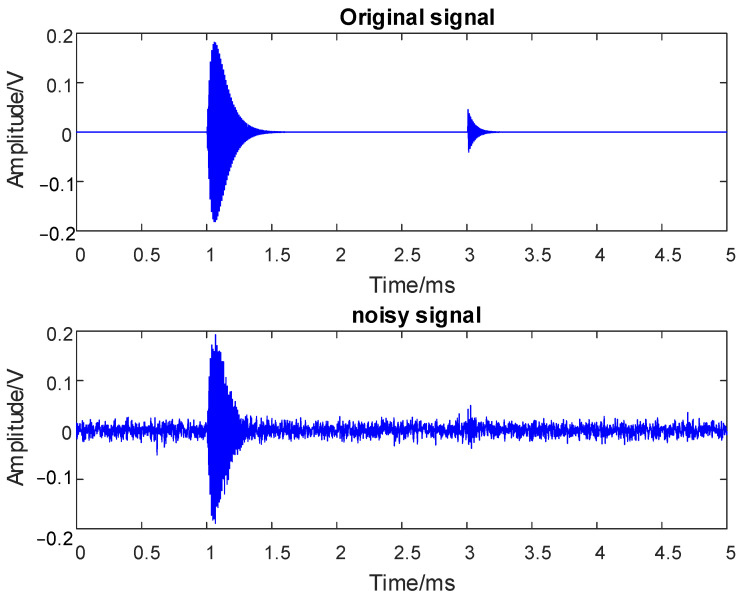
Simulated PD signal.

The simulation is performed on a PC using MATLAB 2020a with the following specifications—CPU: AMD Ryzen 7 5800 H, RAM: 16GB. Based on the parameter settings in Table 2, two PD pulses form the original simulated signal *S*(*t*). There is a large amount of noise interference in the operation site of transformers, among which random white noise interference is the most common type, mainly caused by the thermal noise of transformer windings and relay protection lines. It has similar time and frequency domain characteristics to PD signals. In order to simulate a more realistic transformer PD signal, this paper attempts to add random white noise to the original signal. Considering the real-world circumstances of signals immersed in noise and noise immersed in signals, the white noise with a signal-to-noise ratio of 30 dB is added to *S*(*t*), resulting in a noisy PD signal *Y*(*t*), depicted in Figure 2. It can be seen that the first PD pulse is interfered with by the noise obviously and the second one is completely immersed into the noise and unable to recognize.

**Figure 3:**
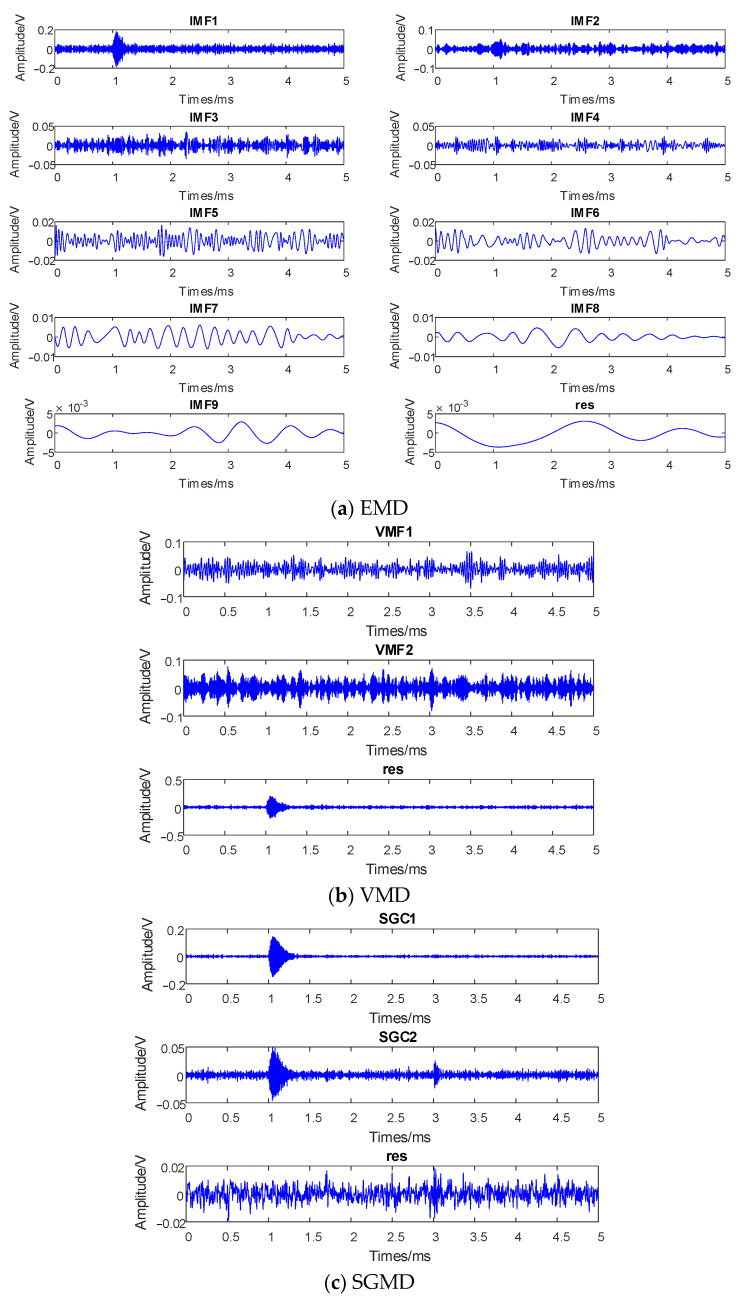
Signal decomposition.

To validate the effectiveness of the proposed algorithm, this paper employs EMD, VMD, and SGMD to decompose the noisy signals separately. The results are illustrated in Figure 3.

From Figure 3a, it can be observed that the original simulated signal is adaptively decomposed into nine IMF components and residuals using EMD. IMF1 exhibits a significant amplitude, indicating that it is a component of a high-frequency PD signal. IMF2-IMF3 can be determined as the background white noise from their amplitudes. However, the remaining IMF components expose the shortcomings of EMD, revealing a clear mode-mixing phenomenon leading to signal distortion. Due to the requirement of presetting the number of decomposition layers in VMD, this study selects the same number of decomposition layers as SGMD. As seen in Figure 3b, after three layers of VMD decomposition, the noise component can be effectively extracted, and the first pulse with a larger amplitude can be successfully identified. VMD overcomes the mode-mixing issue in EMD; however, it fails to identify the second pulse with a smaller amplitude, leading to information loss. Figure 3c illustrates the time-domain diagram using SGMD. It can be observed that SGMD decomposition results in three components, among which SGC1 and SGC2 exhibit higher frequencies and amplitudes. By comparing the period similarity, it can be indicated that the residual represents the random noise components, effectively overcoming the mode-mixing problems. 

## 4. Power Transformer PD Fault Diagnosis Based on SGMD ApEn and Optimized BILSTM

The proposed PD fault diagnostic process for the power transformer is as follows.

(1) Firstly, under laboratory conditions, collect experimental PD signals, including bubble discharge (BD), corona discharge (CD), surface discharge (SD), and floating discharge (FD);
(2) Next, apply SGMD to these PD signals for decomposition. This process breaks down the intricate PD signals into various SGC components, effectively extracting information pertaining to different frequency components;
(3) Subsequently, employ the principle of similarity to select relevant SGC components, and compute their approximate entropy (ApEn) values to serve as quantified features of PD signals;
(4) Finally, utilize the obtained ApEn values as inputs to construct the BILSTM model.

**Figure 4:**
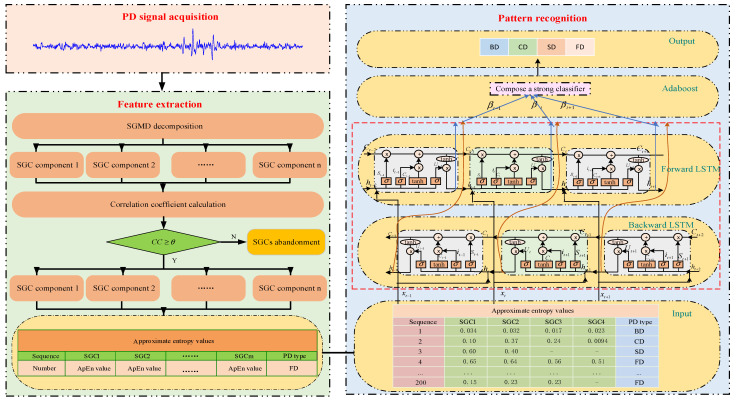
PD fault diagnosis based on SGMD and optimized BILSTM.

Through learning and training, this model can discern distinct characteristic patterns of various PD types, thereby accomplishing the diagnosis of PD signals in transformers. The diagnostic flowchart is illustrated in Figure 4.

### 4.1. PD Data Acquisition

**Figure 5:**
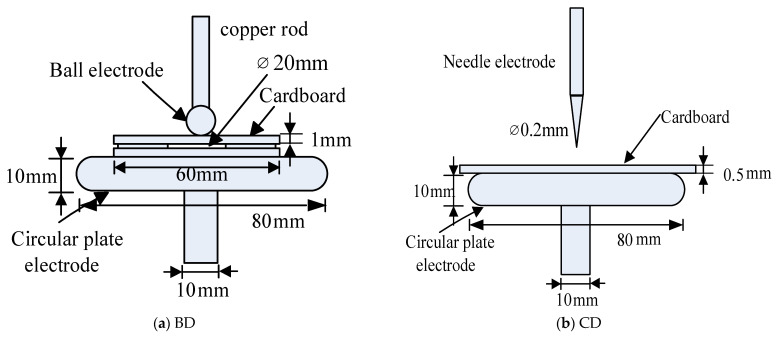

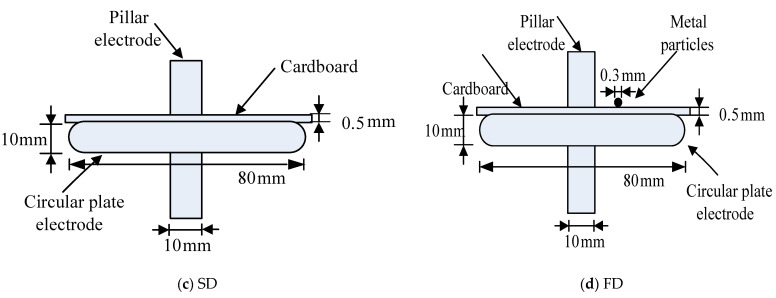
PD physical models.

In the laboratory, four types of PD models are designed, as shown in Figure 5. All circular electrodes have a diameter of 80 mm and a thickness of 10 mm. All PD models are placed in the tank containing transformer oil. 

**Figure 6:**
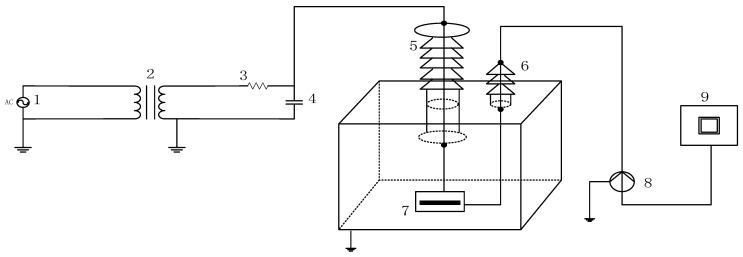
Schematic of PD experiment connections.

PD measurements are conducted in a laboratory-simulated transformer oil tank, and the experimental wiring is shown in Figure 6. The sampling frequency is 15 MHz. The execution standard for PD measurement is IEC 60270.

**Figure 7:**
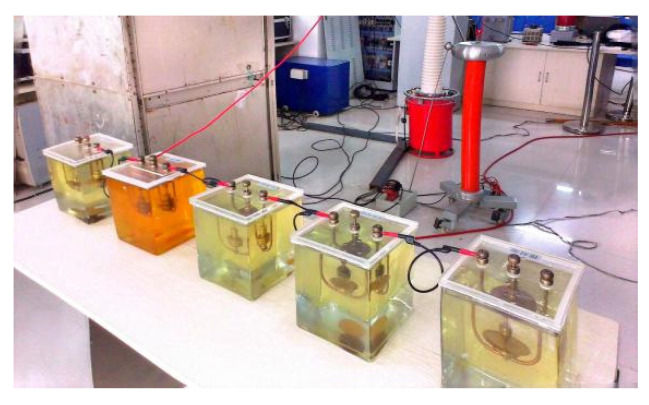
PD models.

In Figure 6, 1 represents the AC power source, 2 is the boosting transformer, 3 is the protective resistor, 4 is the coupling capacitor, 5 is the high-voltage bushing, 6 is the small bushing, 7 is the PD model, 8 is the current sensor, and 9 is the control console. The coupling capacitor is a 500 pF high-voltage coupling capacitor with a withstand voltage of 100 kV, used to couple the PD pulse current generated by the discharge model. The step-up transformer consists of an auto-transformer and a corona-free test transformer. In this experiment, the PD model is placed in a tank filled with oil and grounded through a low-voltage bushing. The pulse current generated on the grounding wire is measured by a current sensor with a detection frequency band of 500 kHz to 16 MHz. The signal was input to the TWPD-2E PD analyzer through a cable for display and storage. The indicators of the analyzer are shown in Table 3. The test conditions for the PD models are shown in Table 4. In this article, four different PD types, BD, CD, SD, and FD, are collected in a laboratory environment as shown in Figure 7. 

**Figure 8:**
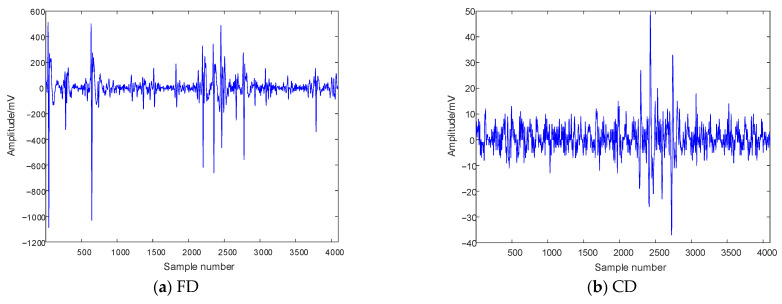

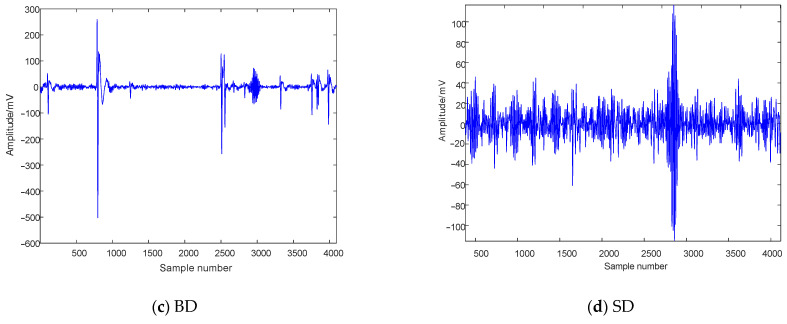
PD experimental signals.

After applying AC voltage externally, the PD model may experience PD within a positive and negative half cycle period, with positive amplitude occurring during the positive half cycle and negative amplitude occurring during the negative half cycle. There are significant differences in the positive and negative half cycle amplitudes of different PD types. The collected PD signals are depicted in Figure 8.

### 4.2. SGMD Decomposition

**Figure 9:**
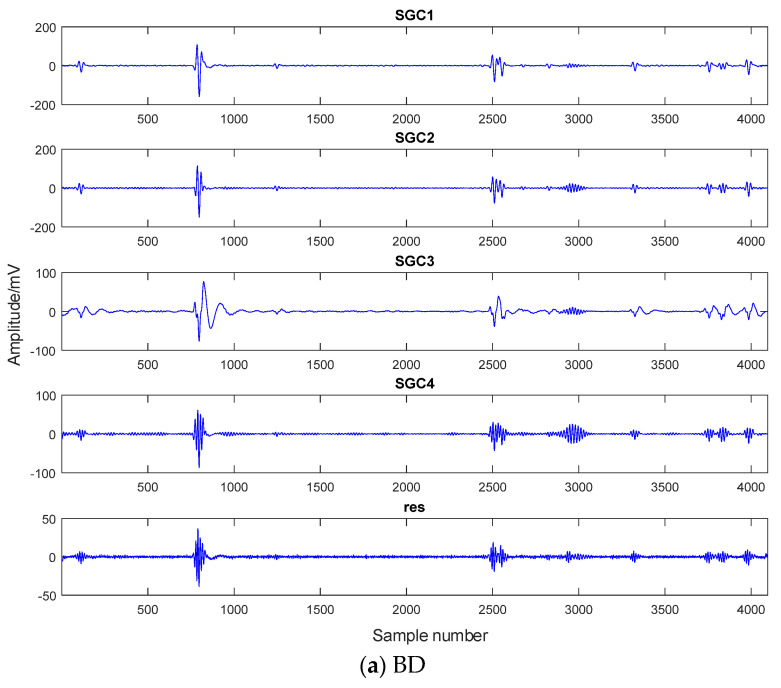

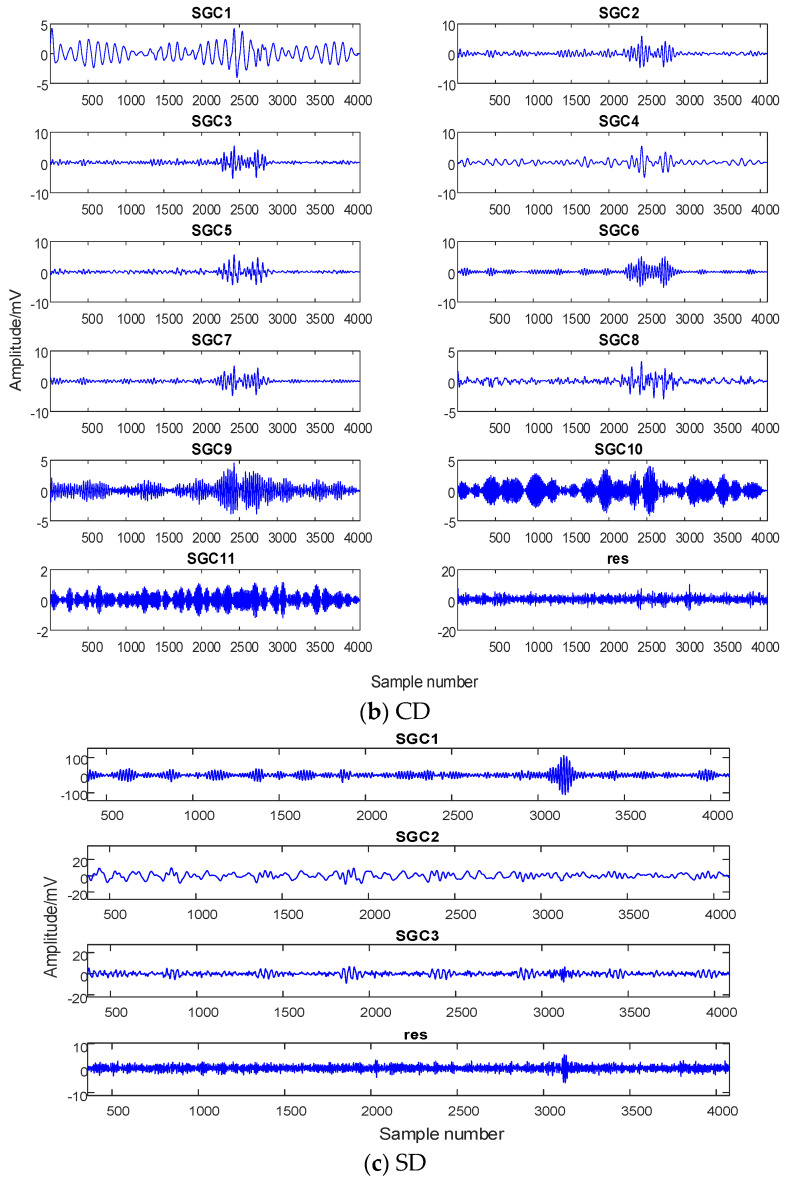

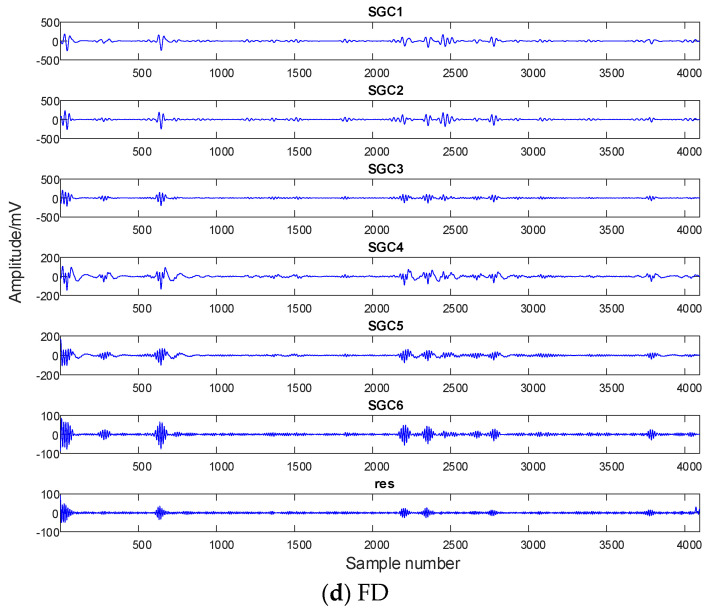
SGMD decomposition.

In this article, the SGMD decomposition is performed on the experimental PD signals. The results are shown in Figure 9.

From Figure 9, it is evident that the SGMD decomposition yields distinct SGC components for different PD types. For instance, a BD signal generates five SGC components, while a CD signal produces twelve SGC components, with four SGC components for SD, and six SGC components and a residual component for FD. The selection of relevant SGC components for subsequent analysis becomes necessary.

### 4.3. Effective SGC Components Selection

To extract the effective components of the PD signals, this study employs a correlation coefficient (*CC*) analysis method [41]. CC is computed between each SGC and the original PD signal consisting of 4096 data points. The definition of CC is as follows.

(3) $$CC=\frac{\sum_{i=1}^{n}\left(x_{i}-\bar{x})(SGC_{i}-\bar{SGC}\right)}{\sqrt{\sum_{i=1}^{n}{\left(x_{i}-\bar{x}\right)}^{2}}\sqrt{\sum_{i=1}^{n}{\left(SGC_{i}-\bar{SGC}\right)}^{2}}}$$

where *x*_i_ represents the original signal, $\bar{x}$ represents the average value of *x*, and *n* represents the number of components of *SGC*. 

**Figure 10:**
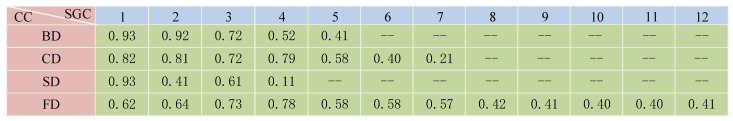
CC values.

The *CC* value for each *SGC* is obtained using Equation (3), as shown in Figure 10. The *CC* value can effectively quantify the similarity between two different time series. Figure 10 displays the similarity between the *SGC*s and the original PD signals.

In order to eliminate the *SGC*s with lower similarity, a threshold *θ* can be preset. If the *CC* value is greater than *θ*, those *SGC*s will be retained as useful components. Otherwise, they will be considered invalid components and removed. The threshold definition in this article is as follows [42].

(4) $$\theta =\sqrt{\frac{\sum_{i=1}^{n}{\left(CC_{i}-\bar{CC}\right)}^{2}}{n}}$$

**Figure 11:**
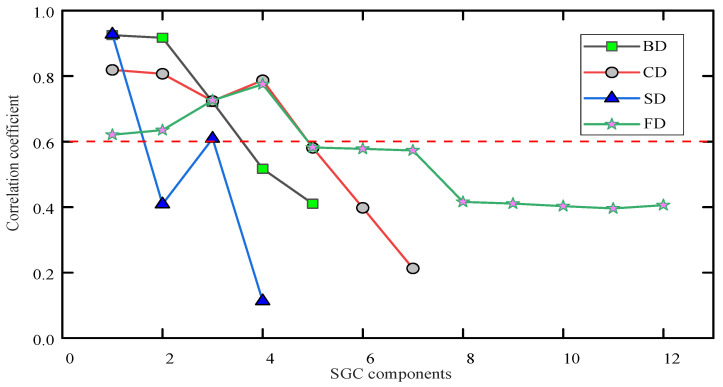
Correlation coefficients varying with different SGC components.

After multiple trials, *θ* is set to 0.6. *CC* values in *SGC* components of different PD types are present in Figure 11.

As shown in Figure 11, the SGCs calculated from different PD types exhibit distinct variations in their CC values. For FD, the CC of the first four SGC components exceeds 0.6, indicating the higher ability to represent prominent signal features. Therefore, the first four SGC components are selected as the main characteristics for FD. Similarly, for CD, the first four SGC components are chosen, while for BD, the first three components are selected, and for SD, the first and third components are kept.

### 4.4. ApEn Calculation

**Figure 12:**
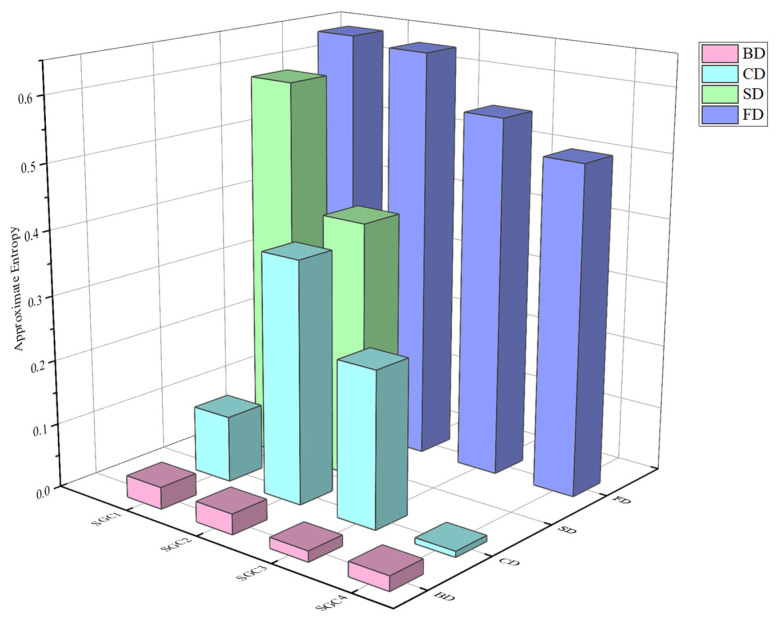
ApEn values for different PD types.

As described in Section 4.3, different SGC components are selected as the significant characteristics for different PD types. To further quantify PD features, this study introduces the approximate entropy for uncertainty analysis of the extracted SGC components. By computing the entropy values of each component, it becomes possible to assess the complexity and irregularity of PD signals. Higher ApEn values indicate a higher level of complexity in the SGC component, suggesting a greater complexity and severity in PD signals. The ApEn values for each SGC component are presented in Figure 12.

**Figure 13:**
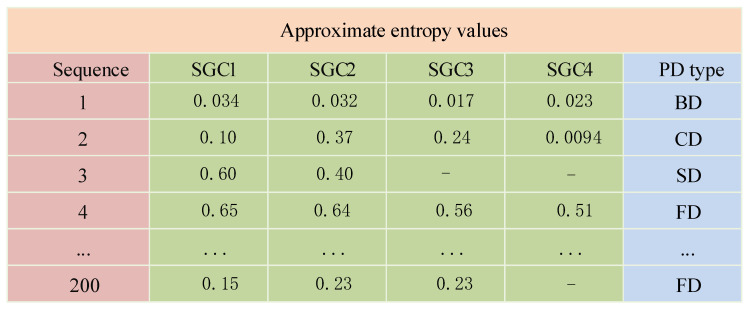
Partial approximate entropy values.

As shown in Figure 12, the effective SGC components of different PD types yield distinct ApEn values. ApEn is able to quantify the complexity of various SGC components; therefore, it can serve as a characteristic parameter for PD signals. By calculating the ApEn values, information about the PD type can be obtained, facilitating subsequent diagnostic analysis. This work collects 50 sets of experimental data for each PD type. The effective SGCs are selected for ApEn calculations. The partial entropy values obtained are shown in Figure 13.

### 4.5. Pattern Recognition

This paper utilizes the entropy values obtained in Section 4.4 as the final PD characteristic parameters. These parameters are fed into the optimized BILSTM model for recognition, thereby achieving the diagnostic results. For each type of PD signal, 15 sets are selected for training and 35 sets for testing.

#### 4.5.1. Selection of BILSTM-Adaboost Parameters

Initially, BILSTM-Adaboost hyper-parameters are initiated through manual experience. By limiting the maximum number of training iterations to 10 and allocating 30% of the population to the explorers, optimized BILSTM hyper-parameters are obtained through GJO optimization, as shown in Table 5.

**Figure 14:**
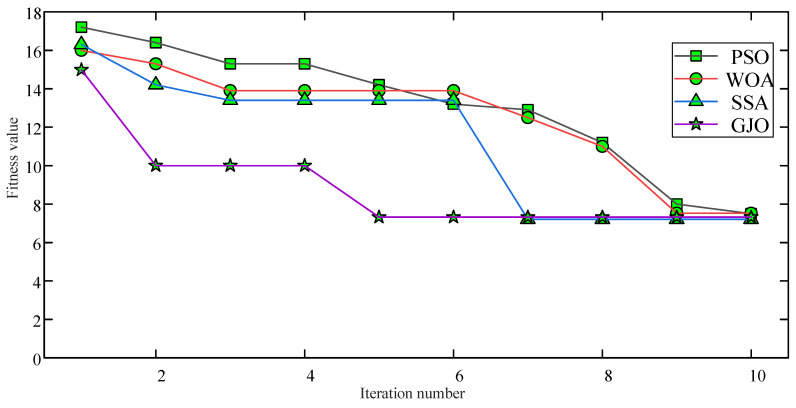
Fitness curves.

**Figure 15:**
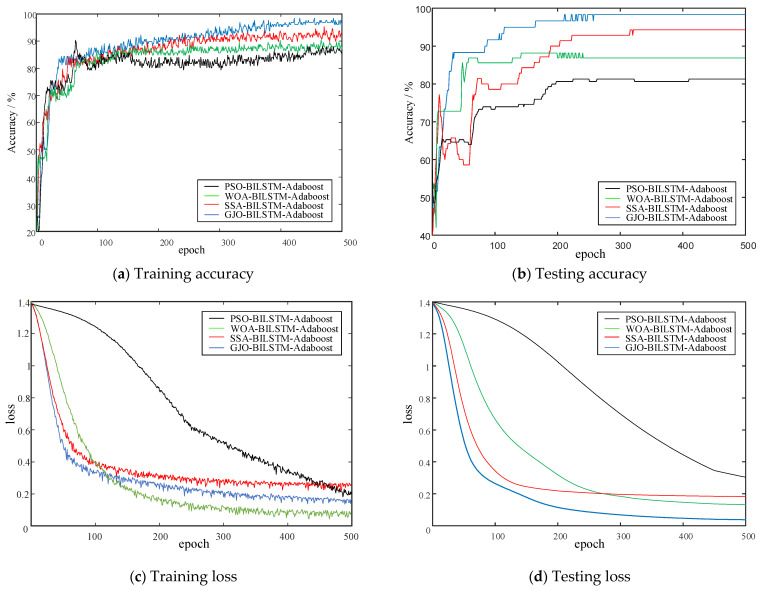
Accuracy and loss.

Using the minimum envelope entropy as the objective function, the population size is set to 20 and the number of iterations is set to 10. Figure 14 shows the fitness curves for optimizing BILSTM hyper-parameters using Particle swarm optimization (PSO), the Whale Optimization Algorithm (WOA), Stochastic Simulated Annealing (SSA), and GJO, separately. The accuracy and loss function obtained from PD diagnosis are shown in Figure 15.

The comparative results in Figure 14 indicate that the optimized BILSTM parameters using GJO leads to faster convergence and requires fewer iterations to stabilize fitness values than other methods. This suggests that GJO exhibits a higher search capability in parameter optimization, making it more efficient at finding optimal solutions.

Figure 15a,b show that the accuracy of GJO-BILSTM-Adaboost is significantly higher than that of the other three optimized classifiers both in training and testing. Figure 15c,d indicate that the GJO-BILSTM-Adaboost has an obvious decrease in both training and testing loss.

#### 4.5.2. Results Analysis

**Figure 16:**
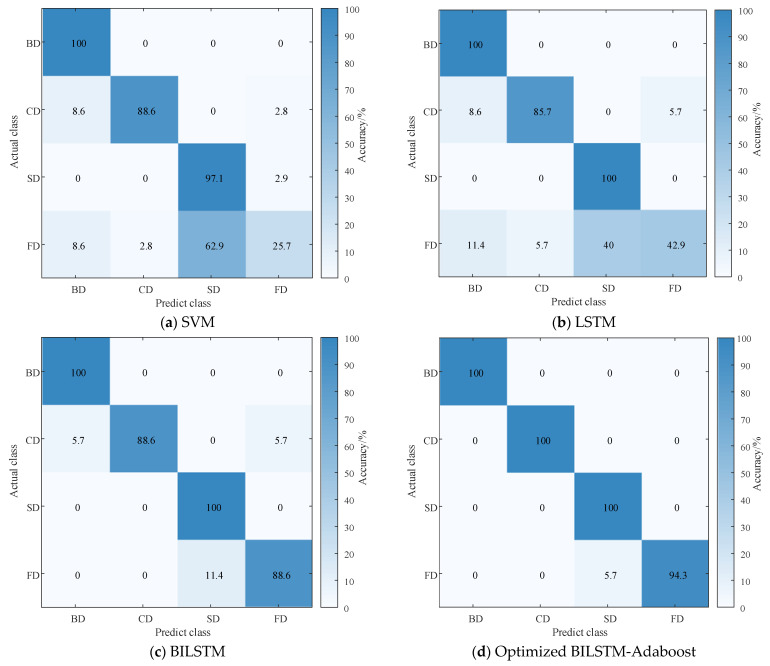
Diagnostic results of different classifiers.

Based on Section 4.5.1, this paper obtains a BILSTM model that has been optimized. The test data are fed into the trained BILSTM model. Additionally, a comparative analysis is conducted with SVM, LSTM, and BILSTM. Parameters of LSTM and SVM are preset in Table 6, where σ is the kernel parameter of RBF and C is the penalty factor in SVM. The diagnostic results are shown in Figure 16. 

As shown in Figure 16, the diagnostic results are presented in the form of a confusion matrix. The main diagonal represents the probability of the model correctly classifying in the classification task, while the rest of the positions represent the misjudgment rate. In Figure 16a, the recognition rate of SVM for FD is extremely low, only 25.4%. In particular, distinguishing between SD and FD is challenging. This suggests that SVM has high data requirements and may suffer from overfitting, leading to poor generalization performance. In Figure 16b, LSTM has improved the overall diagnostic recognition rate; however, misclassification still occurs, especially in the cases of SD and FD. This indicates that LSTM’s performance is sensitive to parameter selection. Figure 16c demonstrates that BILSTM achieves a recognition accuracy of over 85% for each type of PD fault. It correctly identifies BD and SD faults and outperforms traditional SVM and LSTM. Figure 16d reveals that after optimization with GJO, BILSTM achieves better recognition accuracy for all PD faults. This illustrates that GJO, through adaptive search strategies, further enhances the network’s generalization performance.

**Figure 17:**
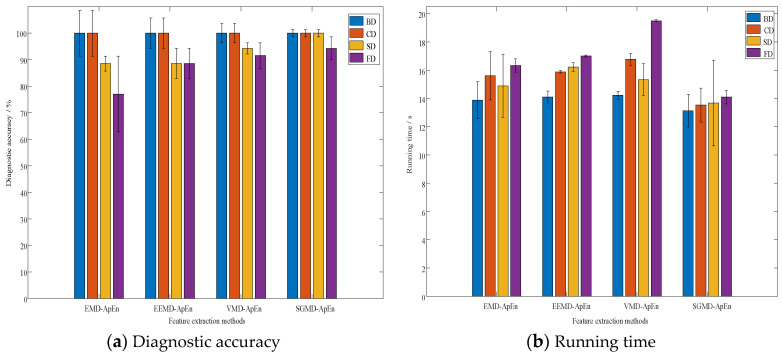
Comparative results.

In order to validate the superiority of the feature extraction method proposed in this paper, the article introduces EMD, EEMD, and VMD methods for PD signal decomposition, after which the ApEn values are calculated. Parameters of EMD, EEMD, VMD, and SGMD are present in Table 7. Nstd is the regularization parameter in EMD and EEMD, and NE is the maximum number of IMFs in EEMD. In VMD, *k* is the number of decomposition layers, alpha is used to control the stability and convergence of the mode, and tol is the calculated tolerance. In SGMD, threshold_corr is the threshold for mode selection and threshold_ne is the threshold for noise evaluation. As PD features, the ApEn values are sent into the optimized BILSTM. The diagnostic accuracy and the algorithm runtime are illustrated in Figure 17.

From Figure 17a, it can be concluded that the results based on EMD-ApEn have obvious misjudgments in SD and FD. This is attributed to the mode-mixing problem in EMD decomposition, where some modes may interfere with each other during PD signal decomposition. The EEMD-ApEn method shows some improvement in the recognition of FD; however, the overall diagnostic performance for SD and FD faults is not satisfying. This is caused by the remaining modal aliasing issues in EEMD, along with the sensitivity to initial conditions. In VMD, a higher diagnostic correctness rate is obtained, indicating that the modal aliasing effect has been suppressed; however, VMD requires manual setting of decomposition layers or modal quantities, relying on a high degree of manual expertise. Improper settings can adversely impact the final diagnostic results. The feature extraction based on SGMD-ApEn achieves a satisfactory diagnostic result with smaller accuracy fluctuations, indicating that SGMD offers high-resolution modal components, aiding in capturing signal details and variations accurately. Figure 17b shows that the SGMD-ApEn method takes the shortest running time with smaller time fluctuations for each PD type. The above results prove that the feature extraction method based on SGMD-ApEn can accurately represent the PD information and shows better performance compared with other methods. 

#### 4.5.3. Imbalanced Data Validation

**Figure 18:**
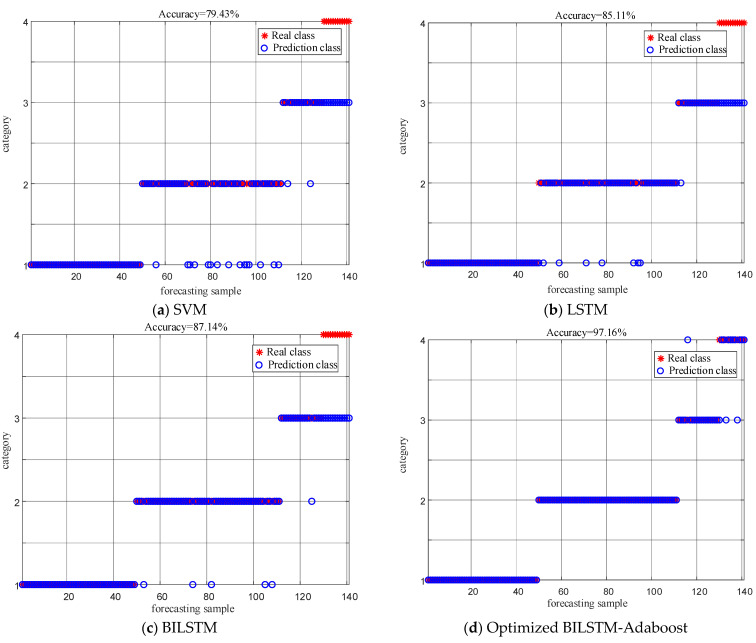
Diagnostic results of different classifiers on imbalanced datasets.

Due to the imbalance problem of different PD types in practical engineering applications, it is necessary to verify the performance of the method proposed in this article in handling imbalanced data. Based on the current statistical analysis of the number of PD faults in transformers, this article sets the data ratios for different PD types as follows: 70 BD faults, 88 CD faults, 25 SD faults, and 17 FD faults. The diagnostic results obtained using different classifiers are shown in Figure 18.

As shown in Figure 18, the GJO-BILSTM-Adaboost classifier introduced in this article demonstrates significant advantages in handling imbalanced data, with a recognition accuracy of up to 97.16% and a decrease of only 1.41% compared to balanced data. Combining BILSTM and Adaboost, the optimized model shows better performance than other methods. Adaboost can improve the diagnostic accuracy of the model with imbalanced data.

#### 4.5.4. Noise Sensitivity Analysis

This article conducts a noise sensitivity analysis for the proposed method. The results of different methods before and after wavelet denoising are compared in Table 8.

It can be concluded from Table 8 that among different signal decomposition methods before and after denoising, SGMD has the smallest running memory and the highest recognition accuracy. The diagnostic accuracy of EMD, EEMD, VMD, and EMD-VMD has been significantly improved after wavelet denoising. It demonstrates that these methods have poor noise-suppression effects. The diagnostic accuracy of SGMD remains nearly unchanged before and after noise reduction, with less noise sensitivity. Moreover, the proposed PD diagnostic model based on SGMD-GJO-BILSTM-Adaboost shows outstanding performance in PD fault diagnosis with a recognition accuracy of 98.57%, obviously superior to other methods.

## 5. Conclusions

This paper proposes a novel method for diagnosing PD faults in power transformers based on SGMD and an optimized bidirectional long short-term memory neural network to improve PD fault diagnostic accuracy. The feature extraction based on SGMD and approximate entropy can quantify the complexity and randomness of PD features and reduce the need for manual parameter tuning, enhancing the computational efficiency. In this study, the GJO optimization algorithm is employed to fine-tune BILSTM hyper-parameters, improving the generalization performance and enhancing the model’s robustness. The extracted PD features are sent into the optimized BILSTM, establishing a novel PD fault diagnostic model. Compared with different feature extraction methods including EMD-ApEn, EEMD-ApEn, and VMD-ApEn, the SGMD-ApEn method takes the shortest running time with smaller time fluctuations and achieves better diagnostic performance. Meanwhile, the optimized BILSTM improves the recognition accuracy of PD faults and outperforms other traditional methods. In addition, the proposed method is also effective for imbalanced data and has lower sensitivity to noise. In the future, the authors will attempt to use more on site data to verify the effectiveness of this method in handling the PD of different transformer models.

## Figures and Tables

**Table 1:** Comparison of different diagnostic methods.

Methods		Advantages	Disadvantages
Feature extraction	Time-frequency domain analysis	Comprehensive and detailed information	Computational complexity, high reliance on experience
	Wavelet transform	Superior multi-scale analytical performance, strong adaptability	High resource demand, complex selection of basis functions
	EMD	Strong nonlinear processing ability	Endpoint effects and modal aliasing
	VMD	Clarity of modal functions, high computational efficiency	Lack of adaptability
Pattern recognition	DBN (Deep Belief Network)	High training efficiency	Computational complexity
	CNN (Convolutional Neural Networks)	High transfer learning ability, convenient parameter sharing	High memory demand, easy to overfit
	RNN	Strong ability to process sequential data	Multiple variants configuration

**Table 2:** PD pulse parameters.

Pulse Number	*K*	*α*_1_	*α*_2_	*τ*	*f_c_*
1	1.0	−1.3	−2.2	0.1	0.2
2	0.05	−1	-	0.05	0.1

**Table 3:** Performance specifications of the analyzer.

Items	Description
Measurement channel	Two independent channels
Detection sensitivity	0.1 pC
Sampling accuracy	12 Bit
Maximum sampling rate	20 MHz
Measurement range	0.1 pC–10,000 nC
Non-linearity error within the full scale	5%
Measurement bandwidth	10 kHz–1 MHz
Test power supply frequency range	50–500 Hz
Power supply	AC 220 V; frequency 50 Hz; power 300 W

**Table 4:** Test conditions of PD models.

Discharge Type	Inception Voltage/kV	Breakdown Voltage/kV	Testing Voltage/kV	Sample Number
BD	5	10	6/7/8	15/20/15
CD	8.8	12	9/10/11	15/20/15
SD	3	10	5/6/7	15/20/15
FD	2	7	3/4/5	15/20/15

**Table 5:** BILSTM-Adaboost hyper-parameters.

Hyper-Parameters	Range	Initial Manual Configuration	GJO Optimized Configuration
the learning rate	0.001~0.01	0.01	0.0035
L2 regularization parameter	0.001~0.01	0.01	0.00013
BILSTM layer	1~50	6	13
the maximum training times	200–1000	500	300
the learning rate decline factor	0.1~1	0.1	0.5

**Table 6:** Parameters of SVM and LSTM.

Algorithms	Parameter Type	Values
SVM	σ	0.28
	C	9.36
LSTM	Input	11
	Output	3
	Hidden layer	12

**Table 7:** Parameters of EMD, EEMD, VMD, and SGMD.

Algorithms	Parameter Type	Values
EMD	Nstd	0.1
EEMD	Nstd	0.1
	NE	100
VMD	K	8
	Alpha	2000
	Tol	1 × 10^−7^
SGMD	threshold_corr	0.95
	threshold_ne	0.01

**Table 8:** Noise sensitivity analysis.

Methods	Before Denoising			After Denoising		
	Running Memory	Running Time	Diagnostic Accuracy	Running Memory	Running Time	Diagnostic Accuracy
EMD-SVM	8472 MB	45.30 s	60.71%	8510 MB	48.72 s	70.00%
EEMD-SVM	8391 MB	40.82 s	64.29%	8436 MB	44.67 s	72.86%
VMD-SVM	8652 MB	50.35 s	69.28%	8720 MB	53.16 s	76.43%
SGMD-SVM	7752 MB	38.96 s	77.86%	7889 MB	40.15 s	77.86%
EMD-LSTM	8348 MB	53.21 s	65.71%	8350 MB	57.34 s	80.00%
EEMD-LSTM	8283 MB	48.73 s	69.28%	8306 MB	56.92 s	80.71%
VMD-LSTM	8963 MB	58.26 s	75.00%	8995 MB	65.00 s	81.43%
SGMD-LSTM	7945 MB	46.87 s	82.14%	7990 MB	49.54 s	81.43%
EMD-BILSTM	8408 MB	54.80 s	70.07%	8435 MB	58.30 s	87.86%
EEMD-BILSTM	8390 MB	50.32 s	77.85%	8419 MB	51.00 s	91.43%
VMD-BILSTM	8896 MB	59.85 s	85.71%	9042 MB	62.52 s	93.57%
SGMD-BILSTM	7889 MB	48.46 s	94.29%	8003 MB	51.25 s	95.00%
EMD-GJO-BILSTM-Adaboost	8584 MB	60.74 s	91.43%	8672 MB	61.35 s	95.71%
EEMD-GJO-BILSTM-Adaboost	8332 MB	63.23 s	94.29%	8499 MB	63.86 s	96.43%
VMD-GJO-BILSTM-Adaboost	8944 MB	65.85 s	96.43%	9076 MB	66.81 s	97.56%
SGMD-GJO-BILSTM-Adaboost	7992 MB	54.46 s	98.57%	8139 MB	55.59 s	98.57%

## Data Availability

The data are not publicly available due to privacy or ethical restrictions.

## List of Acronyms and Symbols

PD	Partial discharge	GJO	Golden jackal optimization
SGMD	Symplectic geometry mode decomposition	FD	Floating discharge
CD	Corona discharge	BD	Bubble discharge
SD	Surface discharge	IMFs	Intrinsic mode functions
SGC	Symplectic geometric components	ApEn	Approximate entropy
EMD	Empirical mode decomposition	VMD	Variational mode decomposition
RNN	Recurrent Neural Networks	LSTM	Long short-term memory
BILSTM	Bidirectional long short-term memory	β	The model weight in BILSTM
WOA	Whale Optimization Algorithm	SSA	Stochastic Simulated Annealing
PSO	Particle swarm optimization	*K*	The signal amplitude
*S*(*t*)	Original simulated PD signal	*Y*(*t*)	Noisy PD signal
α_1_, α_2_	The attenuation parameters	*f_c_*	The oscillation decay frequency
*CC*	Correlation coefficient	*SGC_i_*	The *i*th symplectic geometry mode component
*x* *i*	The original signal	*n*	The number of components of SGC
*θ*	The threshold for CC selection	*σ*	The kernel parameter of RBF
*C*	The penalty factor in SVM	Nstd	The regularization parameter
NE	The maximum number of IMFs	*k*	The number of decomposition layers
tol	The calculated tolerance	threshold_corr	The threshold parameter used for mode selection
threshold_ne	The threshold parameter for noise evaluation		
